# DNMT3A dysfunction promotes neuroinflammation and exacerbates acute ischemic stroke

**DOI:** 10.1002/mco2.652

**Published:** 2024-07-14

**Authors:** Tian‐Jie Lyu, Xin Qiu, Yubo Wang, Ling Zhang, Yalun Dai, Xuechun Wang, Shunying Zhao, Meilin Xiang, Lu Cui, Si Cheng, Yang Liu, Hongqiu Gu, Yong Jiang, Xia Meng, Yilong Wang, Xingquan Zhao, Xianwei Wang, Qian Li, Meng Wang, Yingyu Jiang, Zhe Xu, Xinying Huang, Hao Li, Yongjun Wang, Zixiao Li

**Affiliations:** ^1^ Department of Neurology Beijing Tiantan Hospital Capital Medical University Beijing China; ^2^ China National Clinical Research Center for Neurological Diseases Beijing China; ^3^ Center for Metabolic Disease Research Lewis Katz School of Medicine Temple University Philadelphia Pennsylvania USA; ^4^ Department of Biochemistry and Molecular Biology School of Basic Medical Sciences Capital Medical University Beijing China; ^5^ Clinical Center for Precision Medicine in Stroke Capital Medical University Beijing China; ^6^ Advanced Innovation Center for Human Brain Protection Capital Medical University Beijing China; ^7^ Research Unit of Artificial Intelligence in Cerebrovascular Disease Chinese Academy of Medical Sciences Beijing China; ^8^ Center for Excellence in Brain Science and Intelligence Technology Shanghai Chinese Academy of Sciences Shanghai China; ^9^ Beijing Engineering Research Center of Digital Healthcare for Neurological Diseases Beijing China; ^10^ Chinese Institute for Brain Research Beijing China

**Keywords:** clonal hematopoiesis, DNMT3A, functional outcome, proinflammatory, RG108

## Abstract

Somatic mutations related to clonal hematopoiesis of indeterminate potential (CHIP) are risk factors for stroke. The impact of *DNMT3A*, the most mutated gene in CHIP, on clinical functional outcomes of acute ischemic stroke (AIS) remains unclear. In a well‐characterized cohort of 8524 ischemic stroke patients, we demonstrated that *DNMT3A*‐driven CHIP was significantly associated with neurological disability in these patients. With a stroke mouse model of transient middle cerebral artery occlusion (tMCAO), we demonstrated that DNMT3A protein levels in the brain penumbra increased. The DNMT3A inhibitor RG108 administration amplified neutrophil proliferation in the blood, promoted neutrophil infiltration into the brain penumbra, and exaggerated proinflammatory activation in tMCAO male mice. DNMT3A inhibition also significantly increased infarct volume and worsened neurobehavioral function in tMCAO male mice. In conclusion, *DNMT3A* somatic mutations are associated with worsened neurological disability in some patients with AIS, potentially through increased neutrophil proliferation and infiltration in the ischemic brain region. These findings suggest a possible mechanism for proinflammatory activation and tissue damage in the affected brain tissue, highlighting the need for further research in this area.

## Graphical abstract

•Evaluate the association of *DNMT3A*‐driven clonal hematopoiesis and worse neurological functional disability in ischemic stroke patients in a well‐characterized cohort of 8524 patients with AIS.

•Use a molecular inhibitor of DNMT3A RG108 to confirm that DNMT3A‐dysfunction increases infarct volume and worsens neurobehavioral deficits in a tMCAO mouse model.

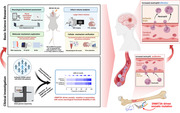

## 1 INTRODUCTION

Ischemic stroke is a leading cause of disability worldwide, with an increasing prevalence in recent years.^1^ Neurological disability after ischemic stroke is a major concern for both patients and caregivers.^2^ However, there remains a lack of effective medications targeting post‐stroke functional recovery.

Clonal hematopoiesis of indeterminate potential (CHIP) is used to describe individuals with a somatic mutation associated with hematologic malignancy in the blood or bone marrow lacking other diagnostic criteria for hematologic malignancy.^3^ A previous study of our large‐scale cohort of stroke patients that was based on a nationwide prospective registry, including 15,166 patients diagnosed with acute ischemic stroke (AIS) or transient ischemic attack (TIA), has shown that the presence of CHIP increases the risk of short‐term recurrent stroke in first‐ever AIS patients.^4^

The gene encoding DNA methyltransferase 3a (*DNMT3A*), the most common acquired mutation causing CHIP, is also important in epigenetic regulation and is thought to play a role in de novo methylation.^5^ The presence of *DNMT3A*‐CHIP is associated with a significantly increased risk of heart failure, ischemic stroke, and mortality, with a 1.45‐fold higher prevalence in atrial fibrillation (AF) patients.^6^ Moreover, the prevalence of *DNMT3A*‐CHIP in AIS patients is notable when compared with healthy controls.^7^ Research has revealed that CHIP carriers, including those with *DNMT3A* mutations, face an elevated risk of hemorrhagic stroke.^8^ As we previously reported, CHIP presence correlates with a higher likelihood of recurrent stroke and other vascular events within three months post‐AIS.^9^ Single‐cell transcriptome analysis in leukocytes from patients with heart failure indicates that *DNMT3A* mutations drive an increase in monocyte inflammatory gene expression, which can lead to enhanced proinflammatory activation and endothelial adhesion upon *DNMT3A* silencing in monocytes.^10^, ^11^

Recently, a cohort research reported that *DNMT3A‐*CHIP is significantly linked to worse initial stroke severity as measured by the NIHSS score and is independently associated with a higher risk of hemorrhagic transformation (OR = 3.31, 95% CI = 1.81−6.07) and functional disability at 90 days post‐stroke (OR = 2.31, 95% CI = 1.17−4.53).^7^ Laboratory evidence suggests that global DNA methylation levels are upregulated in the brains of mice following ischemia‐reperfusion.^12^ However, DNMTs inhibitors or siRNAs were incubated with primary cultured neurons or injected into the mouse brain by intracerebral stereotaxic injection, focusing on their protective effect in neurons in previous studies.^13^, ^14^, ^15^, ^16^, ^17^, ^18^, ^19^

However, despite these intriguing findings, the impact of somatic DNMT3A mutations on the functional outcomes of AIS remains an enigma. It is yet to be determined whether these mutations play a neuroprotective role or exacerbate neuronal damage. Furthermore, the underlying mechanisms driving these effects warrant further elucidation. Thus, we aimed to evaluate the potential role of *DNMT3A* mutation on poststroke neurological disability in a well‐characterized cohort of AIS patients and to explore the underlying mechanism of DNMT3A‐exacerbated neurological disability in mice with ischemia/reperfusion brain injury.

## 2 RESULTS

### 2.1 Characteristics of the study population and detection of *DNMT3A*‐driven CHIP

**FIGURE 1:**
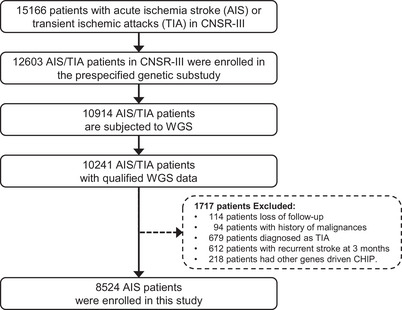
Flowchart of patient enrollment.

A total of 15,166 patients were recruited from August 2015 to March 2018 in the CNSR‐III, which is a nationwide and comprehensive clinical evaluation registry of ischemic stroke or TIA in China based on etiological classification, imaging, and biological markers.^20^ The CNSR‐III comprised 93.3% AIS patients (*n* = 14,146) and 6.7% TIA patients (*n* = 1020). To explore the association of DNMT3A‐driven CHIP with neurological functional disability of ischemic stroke, we included 10,241 AIS/TIA patients with qualified WGS data^21^ in this study. The patients with loss of follow‐up (*n* = 114), history of malignancies (*n* = 94), TIA diagnosis (*n* = 679), recurrent stroke at 3 months (*n* = 612), and CHIP driven by other genes (*n* = 218) were excluded (Figure 1). Finally, 8524 AIS patients were enrolled in this study.

The baseline characteristics of the 8,524 patients are summarized in Table 1. The median age of the patients was 62 years (interquartile range [IQR], 54.0−70.0), and 90 of 8524 patients (1.1%) were identified as *DNMT3A*‐driven CHIP carriers. *DNMT3A*‐driven CHIP carriers were 5 years older than patients without the *DNMT3A* mutation. *DNMT3A*‐driven CHIP carriers were more likely to be female than non‐*DNMT3A* carriers (42.4% vs. 30.2%, *p* = 0.01), which might have led to a significantly lower rate of smoking habits in DNMT3A‐driven CHIP carriers (23.3% vs. 33.3%, *p* = 0.045). Except for coronary heart disease, traditional cardiovascular risk factors, such as a history of AIS/TIA, hypertension, diabetes mellitus, and hypercholesterolemia, were not significantly different between *DNMT3A*‐driven CHIP carriers and non‐*DNMT3A* carriers. The levels of low‐density lipoprotein, cholesterol, and interleukin‐1 receptor antagonist were significantly elevated in *DNMT3A*‐driven CHIP carriers.

**TABLE 1:** Baseline characteristics of the study population.

Characteristics	Total (*n* = 8524)	Non‐*DNMT3A* (*N* = 8434)	*DNMT3A* (*N* = 90)	*P*‐value
Age (y), median (IQR)	62.0 (54.0−70.0)	62.0 (54.0−70.0)	67.0 (60.0−75.0)	<0.0001
Age, *n* (%)				<0.0001
<40	221 (2.6)	221 (2.6)	0 (0)	
40–49	927 (10.9)	925 (11.0)	2 (2.2)	
50–59	2268 (26.6)	2248 (26.7)	20 (22.2)	
60‐69	2927 (34.3)	2897 (34.3)	30 (33.3)	
70–79	1718 (20.2)	1694 (20.1)	24 (26.7)	
≥80	463 (5.4)	449 (5.3)	14 (15.6)	
Female, *n* (%)	2585 (30.3)	2547 (30.2)	38 (42.2)	0.01
BMI (kg/m^2^), mean ± SD	24.8 ± 3.3	24.8 ± 3.3	25.2 ± 4.5	0.63
Current smoking, *n* (%)	2832 (33.2)	2811 (33.3)	21 (23.3)	0.045
Drinking, *n* (%)	1477 (17.3)	1463 (17.3)	14 (15.6)	0.66
Medical history, *n* (%)				
IS/TIA	1948 (22.9)	1933 (22.9)	15 (16.7)	0.16
CHD	877 (10.3)	861 (10.2)	16 (17.8)	0.02
Hypertension	5342 (62.7)	5283 (62.6)	59 (65.6)	0.57
Diabetes mellitus	2025 (23.8)	1999 (23.7)	26 (28.9)	0.25
Hypercholesterolemia	682 (8.0)	671 (8.0)	11 (12.2)	0.14
NIHSS				
Median (IQR)	3.0 (2.0−6.0)	3.0 (2.0−6.0)	4.0 (2.0−6.0)	0.66
TOAST classification, *n* (%)				
LAA	2090 (24.5)	2066 (24.5)	24 (26.7)	0.82
CE	545 (6.4)	537 (6.4)	8 (8.9)	
SAO	1993 (23.4)	1975 (23.4)	18 (20.0)	
SOE	96 (1.1)	95 (1.1)	1 (1.1)	
SUE	3800 (44.6)	3761 (44.6)	39 (43.3)	
Laboratory index, mean ± SD				
hsCRP	7.6 ± 26.3	7.5 ± 26.3	9.3 ± 26.9	0.35
HGB	141.5 ± 16.9	141.6 ± 16.9	138.4 ± 16.0	0.08
WBC	7.3 ± 2.3	7.3 ± 2.3	7.2 ± 2.2	0.69
NEUT	4.9 ± 2.3	4.9 ± 2.2	5.6 ± 6.1	0.44
TG	1.6 ± 0.9	1.6 ± 0.9	1.6 ± 0.8	0.37
LDL	2.4 ± 1.1	2.4 ± 1.1	2.8 ± 1.3	0.01
HDL	1.0 ± 0.3	1.0 ± 0.3	1.0 ± 0.3	0.03
CHOL	4.1 ± 1.2	4.1 ± 1.2	4.5 ± 1.4	0.01
IL‐6	4.3 ± 4.5	4.3 ± 4.5	4.5 ± 4.5	0.31
IL‐1RA	481.8 ± 505.1	480.8 ± 504.7	576.0 ± 537.8	0.01

Abbreviations: BMI, body mass index; CE, cardioembolic; CHD, coronary heart disease; CHOL, cholesterol; HDL, high‐density lipoprotein; HGB, hemoglobin; hsCRP, high sensitivity C‐reaction protein; IL‐1RA, interleukin‐1 receptor antagonist.; IL‐6, interleukin‐6;IQR, interquartile range; IS, ischemic stroke; LAA, large‐artery atherosclerosis; LDL, low‐density lipoprotein; NEUT, neutrophil absolute value; NIHSS, National Institutes of Health Stroke Scale score; SAO, small‐ artery occlusion; SOE, stroke of other determined etiology; SUE, stroke of undetermined etiology; TG, triglyceride; TIA, transient ischemic attack; TOAST, the Trial Org 10172 in Acute Stroke Treatment; WBC, absolute white blood cell.

### 2.2 *DNMT3A*‐CHIP is associated with neurological functional disability in acute ischemic stroke

**FIGURE 2:**
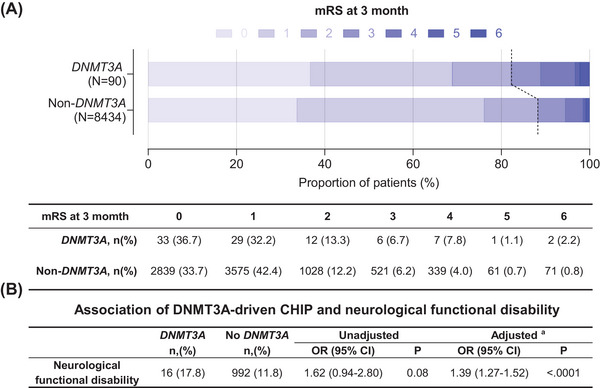
*DNMT3A*‐driven CHIP was associated with neurological functional disability. (A) The proportion of patients with a 3‐month mRS score with a range of 0−6 driven by DNMT3A mutation status. mRS, modified Rankin scale. (B) Association of DNMT3A‐driven CHIP with neurological functional disability. Adjusted^a^ Logistic regression model was adjusted for inverse probability of treatment weighted (IPTW) with age, sex, BMI, smoking, drinking, history of the disease (hypertension, diabetes mellitus, hyperlipidemia, coronary heart disease), admitting NIHSS, and TOAST classification. Neurological functional disability, defined as a modified Rankin scale (mRS) score 3−6; OR, odds ratio; 95% CI, 95% confidence interval.

**FIGURE 3:**
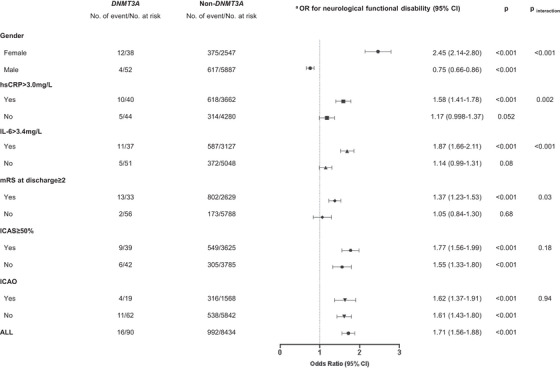
Associations between DNMT3A‐driven CHIP and neurological functional disability at 3 months in selected subgroups. A logistic regression model was adjusted for the inverse probability of treatment weighted (IPTW) and age, sex, BMI, smoking, drinking, history of the disease (i.e., hypertension, diabetes mellitus, and hyperlipidemia), TOAST classification, and NIHSS score. No. of event means the number of patients with neurological functional disability. OR, odds ratio; 95% CI, 95% confidence interval; hsCRP, high‐sensitivity C‐reactive protein; IL‐6, interleukin 6; ICAS, intracranial atherosclerotic stenosis; ICAO, intracranial atherosclerotic occlusion.

During a follow‐up period of 3 months, we observed a higher rate of neurological functional disability in *DNMT3A*‐driven CHIP carriers than that in non‐*DNMT3A* carriers (Figure 2A). Multivariable analysis showed that the odds ratio (OR) with a 95% confidence interval (CI) of *DNMT3A*‐driven CHIP for risk of neurological functional disability was 1.39 (1.27−1.52; Figure 2B). The associations between *DNMT3A*‐driven CHIP and neurological functional disability in the prespecified subgroups are shown in Figure 3. The effect of *DNMT3A*‐driven CHIP on functional disability was adjusted by sex, baseline high‐sensitivity C‐reactive protein (hsCRP) levels, baseline interleukin‐6 (IL‐6) levels, and the modified Rankin scale (mRS) score at discharge. *DNMT3A*‐driven CHIP increased the risk of neurological functional disability only in females (OR, 2.45; 95% CI, 2.14−2.80; *p*_interaction _< 0.001), patients with baseline hsCRP > 3.0 mg/L (OR, 1.58; 95% CI, 1.41−1.78; *p*_interaction _= 0.002), baseline IL‐6 > 3.4 mg/L (OR, 1.87; 95% CI, 1.66−2.11; *p*_ _< 0.001), and patients with mRS ≥ 2 at discharge (OR, 1.37; 95% CI, 1.23−1.53; *p*_interaction _= 0.03).

### 2.3 The DNMT3A inhibitor RG108 significantly increased infarct volume and worsened neurobehavioral deficits in mice with transient middle cerebral artery occlusion

**FIGURE 4:**
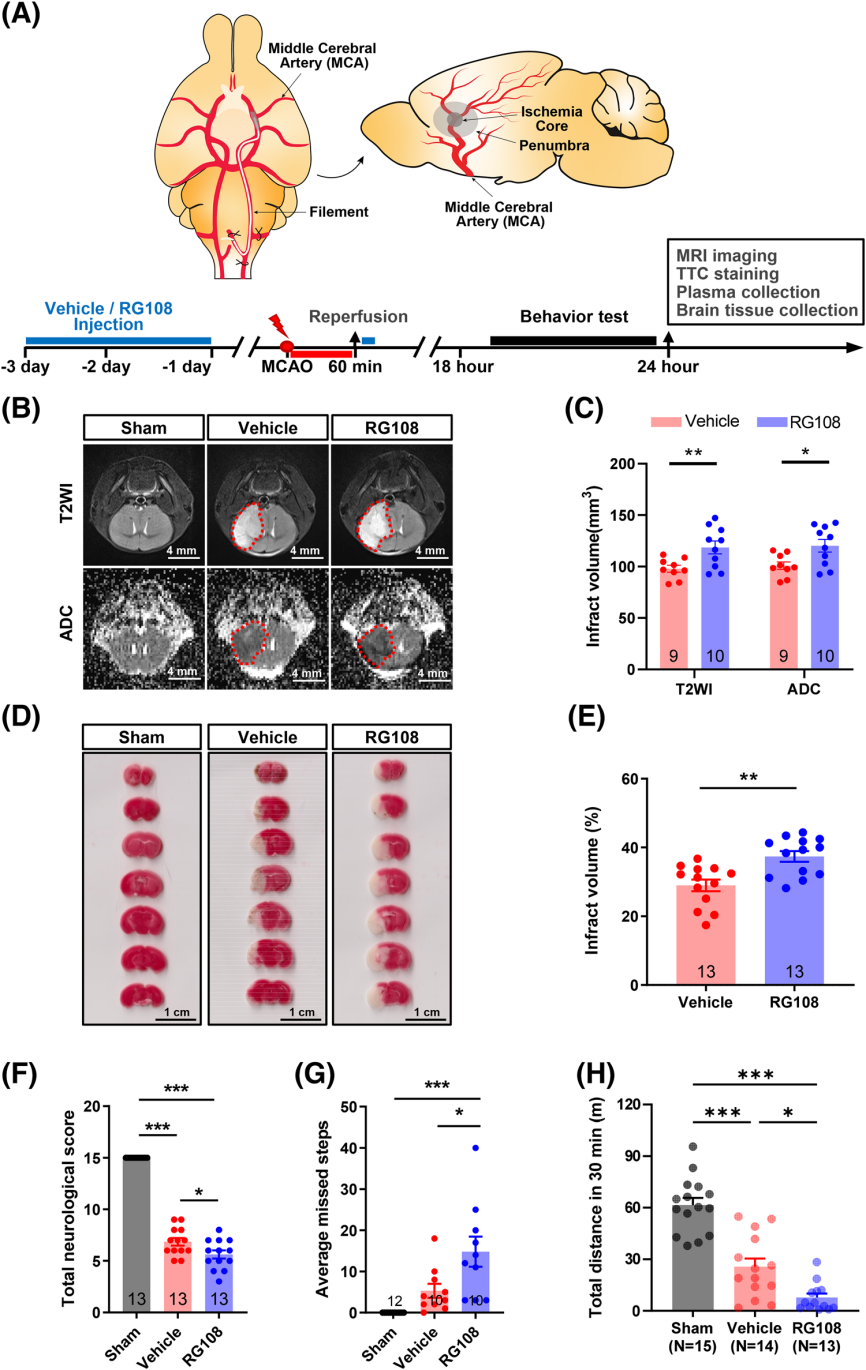
Inhibition of DNMT3A function resulted in larger infarct volumes and worse neurological outcomes in mice. (A) Schematic of experimental study design. (B) Representative T2‐weighted images (T2WI) and ADC images with stroke lesions highlighted by red outline for each group. (C) Quantification of infarct volume based on MRI for each group. (Vehicle, (*n* = 9; RG108, *n* = 10). (D) Coronal brain sections by TTC staining for each group. (E) Quantification of infarct volume based on TTC staining for each group. (vehicle, *n* = 13; RG108, *n* = 13). An independent *t*‐test was used. (F–H) Evaluation of neurological deficits using a modified Garcia test (Sham, *n* = 13; Vehicle, *n* = 13; RG108, *n* = 13), the foot misplacement apparatus (Sham, *n* = 12; Vehicle, *n* = 10; RG108, *n* = 10), and the open field test (Sham, *n* = 15; Vehicle, *n* = 14; RG108, *n* = 13). One‐way analysis of variance (ANOVA) with Tukey's multiple comparisons post hoc test were used. *p < 0.05, **p < 0.01, ***p < 0.001, All data were expressed as mean ± SEM with scatter plots. Scale bar, as indicated.

To explore the potential mechanisms underlying the effect of *DNMT3A* mutation on neurological functional disability in AIS, the function of DNMT3A was inhibited using the small‐molecule compound SGI1027^22^ or RG108 (10 mg/kg)^23^ in mice with transient middle cerebral artery occlusion (tMCAO; Figure 4A). The Preliminary experiment results show that RG108 has superiority over SGI‐1027 in DNMT3A functional inhibition (Figures S1 and S2A). RG108 is recognized as a specific inhibitor of both DNMT3A and DNMT1.^13^, ^17^ At 24 h post‐tMCAO, we assessed infarct volume using T2‐weighted imaging (T2WI), apparent diffusion coefficient (ADC) parameters of magnetic resonance imaging (MRI; Figure 4B), and 2,3,5‐triphenyltetrazolium chloride (TTC) staining (Figure 4D). Our results showed a significant approximately 20% increase in infarct volume in the RG108 group as compared with the vehicle group (T2WI: 118.6 mm^3^ vs. 97.96 mm^3^; ADC: 120.2 mm^3^ vs. 101.0 mm^3^; TTC: 37.39% vs. 28.99%; Figure 4C, E). During 18–24 h post‐tMCAO, three different behavioral tests were used to assess neurological deficits after stroke. First, we scored neurological function using the modified Garcia test,^24^, ^25^ which showed that the RG108 group had a lower mean neurological score than the vehicle group (5.62 vs. 6.85, *p* < 0.05, Figure 4F). Second, the foot misplacement apparatus (FMA) approach^26^, ^27^ showed that mice treated with RG108 tended to have more average missed steps (14.8 vs. 5.3, *p* < 0.05, Figure 4G). Third, an open‐field test was used to assess the motor activity of rodents. The RG108 and vehicle groups displayed a significantly decreased total distance compared with the sham group (Figure 4H). Besides, the RG108 group has a decreased mean total distance compared with the vehicle group (777.2 vs. 2561.6, *p* < 0.05; Figure 4H).

**FIGURE 5:**
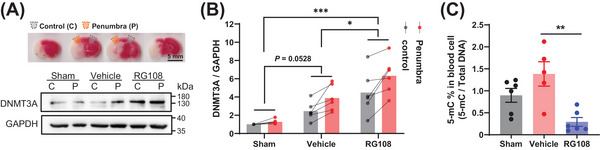
RG108 treatment resulted in DNMT3A inhibition, which referred to the decrease of the 5‐mc level of total DNA. (A) Brain section with penumbra (upper) and western blot for DNMT3a in the infarct penumbra and contralateral region (lower). (B) Quantification of the western blot in panel A (Sham, *n* = 6; Vehicle, *n* = 6; RG108, *n* = 6). Two‐way ANOVA analysis with Tukey's multiple comparisons post hoc test was used. The differences within groups between the control and the Penumbra regions: Sham (*p* = 0.8847), Vehicle (*p* = 0.0106), and RG108 (*p* = 0.0014). (C) DNA methylation level of DNA 5‐mC with the peripheral blood cell using ELISA (Sham, *n* = 6; Vehicle, *n* = 5; RG108, *n* = 6). One‐way ANOVA with Tukey's multiple comparisons post hoc test was used. *p < 0.05, **p < 0.01, ***p < 0.001. All data were expressed as mean ± SEM with scatter plots. Scale bar, as indicated.

RG108 treatment for DNMT3A inhibition resulted in an expansion of infarct volume and deterioration of neurological deficits after cerebral ischemia in mice. Brain tissue and peripheral blood were collected to verify the molecular effect of RG108 on DNMT3A. We quantified DNMT3A using western blotting in tissues of the ischemic penumbra (the area adjacent to the infarct in the 11 o'clock direction of the brain slice) and the control area (the contralateral side of the penumbra, the 13 o'clock direction of the brain slice; Figure 5A). Compared with the sham group, the DNMT3A level was significantly increased in the control area and penumbra in mice with tMCAO, regardless of whether they received RG108. However, RG108 further elevated the DNMT3A level in the penumbra area compared with the vehicle group (Figure 5A, B; Figure S2B). ELISA of peripheral blood cells showed that 5‐methylcytosine (5‐mC) in the RG108 group was significantly lower than that in the vehicle group, which could be due to DNMT3A function loss (Figure 5C). Taken together, cerebral ischemia increased DNMT3A levels in the brain, particularly in the penumbral area. RG108 inhibits DNMT3A function, which leads to a compensatory increase in DNMT3A protein expression.

### 2.4 DNMT3A inhibition boosts neuroinflammation after ischemic stroke

**FIGURE 6:**
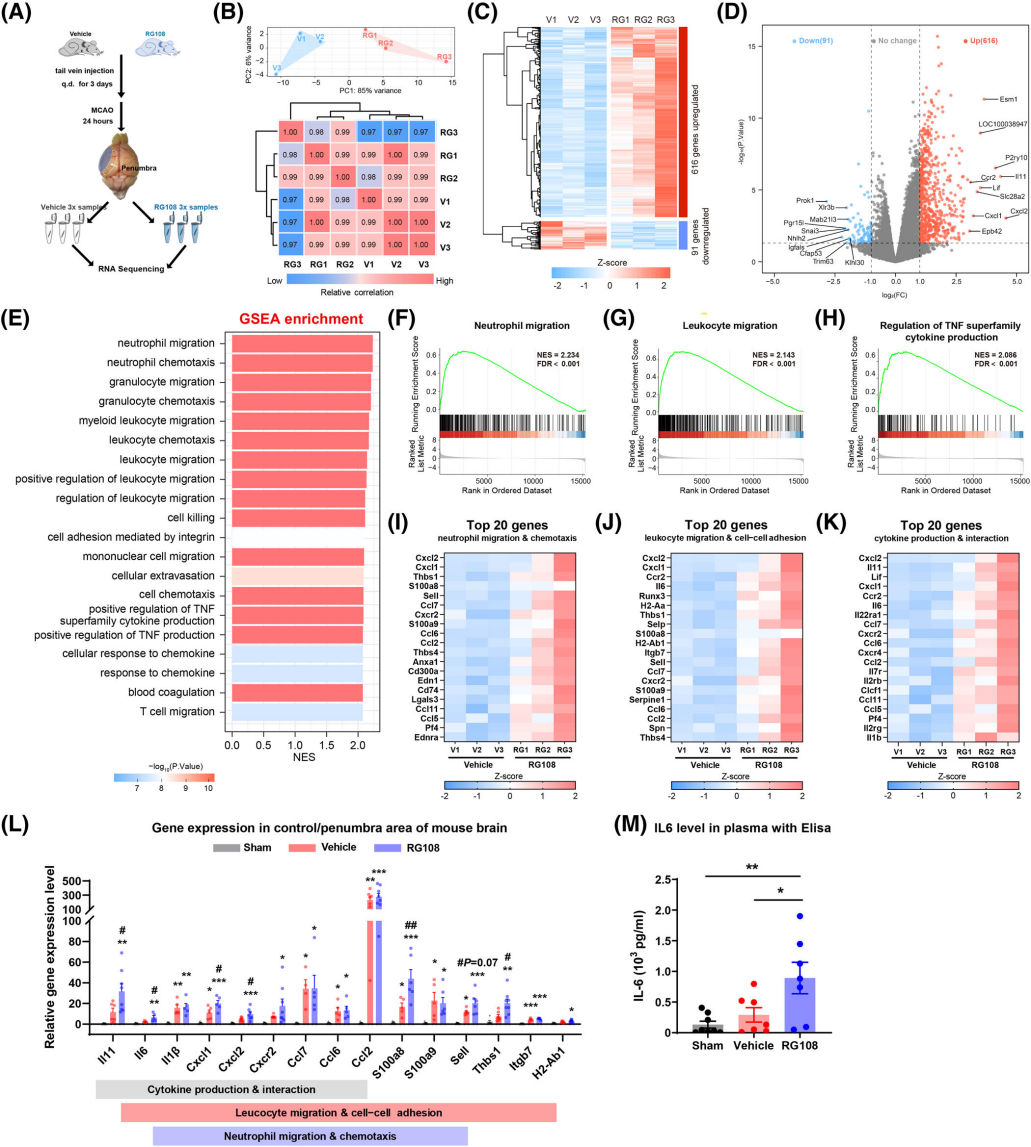
Transcriptome analysis of brain penumbra area in mice with RG108 treatment. (A) Schematic strategy for drug injection, penumbra tissue collection, and RNA sequencing after ischemic stroke. (B) Principal component analysis (upper) and correlation analysis (lower) showed the overall transcriptomic similarity of vehicle group (V) or RG108 group (RG). PC, principal component. (C) The hierarchical heatmap showed the differentially expressed genes (DEGs) in comparison to the RG108 vs. vehicle group. (D) Volcano plots showed the DEGs of the RG108 group as compared with the vehicle group; Red spots indicated the upregulated genes (fold change > 2 and adjusted *p‐*value < 0.05). Blue spots indicated the downregulated genes (fold change < 2 and adjusted *p*‐value < 0.05); The labeled gene indicated the top 10 genes by fold change. FC, fold change. (E) GSEA enrichment analysis was performed with DEGs in the RNA‐sequencing data. The top 20 pathways ranked by NES score were shown. *p*‐values were shown as legends indicated. (F–H) The three representative pathways upregulated in the RG108 group were presented. Genes were preliminarily ranked according to the fold change values, and each vertical black bar represented one gene and the corresponding position in the ranked gene list. NES, normalized enrichment score; FDR, false discovery rate. (I–K) The three most potential signal pathways, which were combined with GO terms, KEGG and GSEA pathways, included neutrophil migration & chemotaxis (I), leucocyte migration & cell−cell adhesion (J), and cytokine production & interaction (K). The corresponding heatmaps show the top 20 DEG expressions in the indicated pathway in both groups with Z‐score normalized. (L) RT‐qPCR validation of gene expression IN the poststroke penumbra cortex (*n* = 5−8 per group). One‐way ANOVA with Holm‐Sidak's multiple comparisons post hoc test was used for each gene. (M) ELISA analysis of IL‐6 with the peripheral blood plasma (Sham, *n* = 8; Vehicle, *n* = 8; RG108, *n* = 7). One‐way ANOVA with Tukey's multiple comparisons post hoc test was used. *p < 0.05, **p < 0.01, ***p < 0.001, as compared with Sham group; #*p *< 0.05, ##*p *< 0.01, as compared with Vehicle group. All data were expressed with mean ± SEM for individual plots.

To explore the underlying mechanism of DNMT3A inhibition, we performed a transcriptomic analysis of the ischemic penumbra cortex of mice treated with RG108 or vehicle (Figure 6A). Bulk RNA‐sequencing was used on ischemic penumbra tissues harvested from mice 24 h post‐tMCAO. We processed samples from six mice, with three serving as a vehicle group and three treated with RG108. Principal component analysis showed that the RG108 group differed dramatically from the vehicle group, and there was a relatively small within‐group variability in the transcriptome. Meanwhile, the correlation analysis of gene expression between samples showed similar results to those of the principal component analysis (Figure 6B). We found 616 differentially expressed genes (DEGs) upregulated (fold change > 2 and adjusted *p*‐value < 0.05) and 91 DEGs downregulated (fold change ← 2 and adjusted *p*‐value < 0.05) in the RG108 group compared with the vehicle group (Figure 6C, D; Tables S3 and S4). Upregulated DEGs were used for biological pathway analysis based on gene ontology (GO) enrichment, GO networks, and the Kyoto Encyclopedia of Genes and Genomes (KEGG). RG108 is involved in proinflammatory biological processes, including leukocyte migration and cell–cell adhesion, cytokine production and interaction, extracellular structure and matrix organization, granulocyte/neutrophil migration, and cell–cell adhesion (Figure S3A–D, Tables S5 and S6).

### 2.5 Neutrophil migration and chemotaxis are enhanced by DNMT3A inhibition in ischemic stroke

To avoid the omission of some important but less obviously altered genes in GO and KEGG enrichment analyses, gene set enrichment analysis (GSEA) was performed with all the genes in the experiment.^28^ GSEA showed that the top pathways included neutrophil/granulocyte/leukocyte migration and chemotaxis, and positive regulation of tumor necrosis factor superfamily cytokine production, which were similar to the results of GO terms and KEGG pathway enrichment analysis (Figure 6E–H, Tables S7). According to pathway analysis, three pathways were mainly enriched: neutrophil migration and chemotaxis, leukocyte migration and cell–cell adhesion, and cytokine production and interaction. The expression of the top 20 DEGs is shown in the corresponding heatmaps (Figure 6I–K). To further verify the results of these enrichment analyses, we performed quantitative real‐time PCR and ELISA for a subset of the top DEGs in the indicated pathway. Compared with the sham group, nearly all genes related to the three pathways were significantly upregulated in the poststroke brain. On the other hand, four genes (*Il11*, *Il6*, *Cxcl1*, and *Cxcl2*) of the cytokine production and interaction pathway, six genes (*Il6, Cxcl1, Cxcl2, S100a8, Sell*, and *Thbs1*) of leukocyte migration and cell−cell adhesion, and four genes (*Cxcl1, Cxcl2, S100a8*, and *Sell*) of neutrophil migration and chemotaxis were significantly upregulated in the RG108 group compared with the vehicle group (Figure 6L). As expected, the level of the proinflammatory cytokine IL‐6 in the serum of the RG108 group was also higher than that in the vehicle group (Figure 6M).

### 2.6 Increased neutrophils in the peripheral blood and ischemic hemisphere with DNMT3A inhibition

**FIGURE 7:**
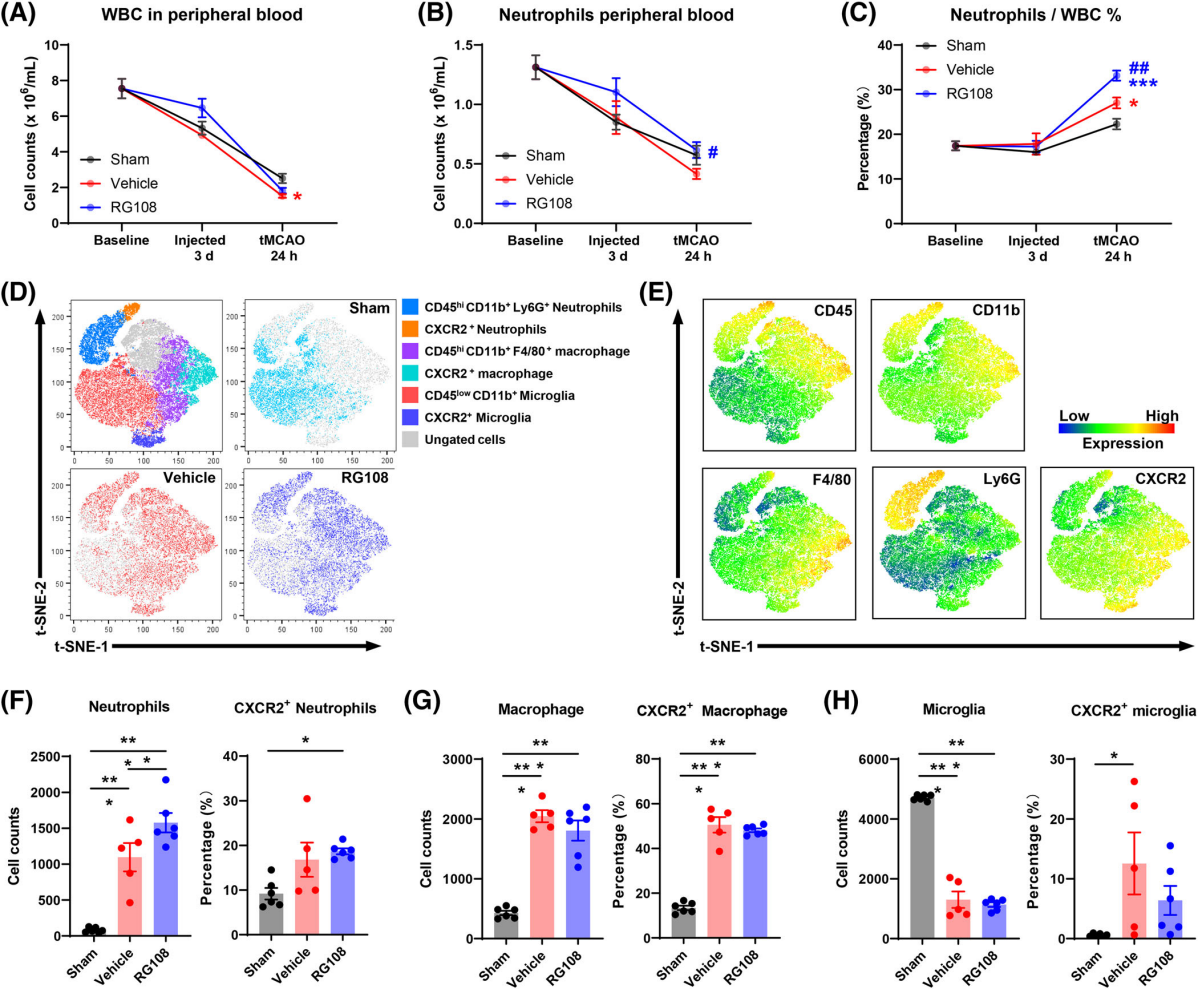
DNMT3A inhibition promotes peripheral blood neutrophil proliferation and infiltration into the ischemic hemisphere after ischemic reperfusion. (A−C) Peripheral hematological changes in mice with different treatments at different time points of untreated (naïve), 3 days after continuous i.p. administration (injected 3 days), 24 h after tMCAO (tMCAO 24 h). White blood cell count (WBC) was shown in (A), neutrophil cell count in peripheral blood in (B), and the percentage of neutrophils in white blood cells in (C). *n* = 8 mice for the time point of baseline, *n* = 8−15 mice per group for the latter two‐time points. two‐way ANOVA analysis with Tukey's multiple comparisons post hoc test was used. * is for Sham group and # is for Vehicle group. (D) Myeloid cells in the poststroke brain were assessed by flow cytometry. *t*‐SNE map of CD45^+^CD11b^+^ myeloid cells concatenated with 5500 cells per sample pooled from all samples in the three groups (top left). Each dot represents a single cell. The colors were coded by their cell type (top left) or experimental group (Sham, vehicle, and RG108). (E) Expression of the markers used for cell clusters for each single cell was overlaid on the t‐SNE map. The color gradient was coded by the marker's intensity. (F−H) We measured the cell count and percentage of CXCR2^+^ activated myeloid cells including neutrophils (F), macrophage (G), and microglia (H). The manual gating measure is shown in Figure S4. Sham, *n* = 6; Vehicle, *n* = 5; RG108, *n* = 6. One‐way ANOVA with Tukey's multiple comparisons post hoc test was used. *p < 0.05, **p < 0.01, ***p < 0.001. All data were expressed as mean ± SEM with individual plots.

To further validate the results of RNA sequencing, we tested the immune response in the peripheral blood and ischemic hemisphere. First, we performed peripheral hematological parameter analysis at three different time points: baseline, 3 days after continuous administration (tMCAO 0 h), and 24 h after tMCAO. White blood cell (WBC, Figure 7A) and neutrophil (Figure 7B) counts continuously decreased in all three groups (sham, vehicle, and RG108). At 24 h after tMCAO, compared with the vehicle group, the RG108 group had many more neutrophils (Figure 7B). In addition, the percentage of neutrophils comprising the WBC increased from 27.03% to 33.16% (*p* < 0.01, Figure 7C). Therefore, DNMT3A inhibition promotes peripheral blood neutrophil proliferation after ischemic reperfusion.

Based on these results, we tested myeloid cells in the ischemic hemisphere 24 h after tMCAO by flow cytometry,^29^, ^30^ and CD45^+^CD11b^+^ myeloid cells were concatenated after manual gating to perform subclustering with high‐dimensional analysis (Figure 7D, E; Figure S4). CD11b^+^CD45^low^ microglia comprised the largest population of immune cells in the brain (Figure 7D). Compared with the sham group, the vehicle and RG108 groups had more infiltrated CD45^+^CD11b^+^Ly6G^+^ neutrophils and CD45^+^CD11b^+^Ly6G^−^F4/80^+^ macrophages in the ischemic hemisphere, including increased CXCR2^+^ activated cells^31^, ^32^, ^33^ (Figure 7D–G). The vehicle and RG108 groups had fewer microglia and more CXCR2^+^‐activated microglia (Figure 7H). In addition, compared with the vehicle group, the RG108 group had much more infiltrated neutrophils (Figure 7F) than macrophages (Figure 7G). However, CXCR2^+^‐activated neutrophils and macrophage cells did not show differences between the vehicle and RG108 groups (Figure 7F, G). Taken together, DNMT3A inhibition promotes the infiltration of neutrophils into the ischemic hemisphere after ischemic reperfusion. To explore proliferation gene expression in neutrophils, we have conducted additional flow cytometry analysis of Ki67 expression in neutrophils from peripheral blood and ischemic brain tissue in tMCAO model mice. The results show that there are more Ki67‐positive neutrophils both circulating and infiltrating into brain tissue with RG108 treated (Figure S5A–D).

## 3 DISCUSSION

Our study suggests that somatic mutations in *DNMT3A* are significantly associated with poor short‐term neurological functional outcomes after stroke, especially in the subgroup of patients with higher hsCRP or IL‐6 levels at baseline and mRS scores at discharge. Meanwhile, animal experiments have demonstrated that inhibition of DNMT3A could lead to poor functional outcomes in tMCAO mice, which might be due to hyperactivation of proinflammatory processes through neutrophil proliferation in the blood and infiltration into the penumbra of the ischemic brain.

Age‐related somatic mutations in *DNMT3A*‐driven CHIP are significantly associated with the progression and poor prognosis of chronic heart failure.^34^ Bhattacharya et al.^35^ demonstrated that mutated *DNMT3A* is associated with the occurrence of hemorrhagic but not ischemic stroke.^35^ However, we recently reported that the presence of *DNMT3A‐*driven CHIP increases the risk of short‐term recurrent stroke in first‐ever AIS patients.^4^ In this study, we also found that the *DNMT3A* mutation increased the risk of short‐term neurological functional disability. Our results can be validated in another cohort research, which reported that *DNMT3A‐*CHIP is significantly linked to worse initial stroke severity as measured by the NIHSS score and a higher risk of hemorrhagic transformation and functional disability at 90 days poststroke.^7^ In our study, due to the WGS data being sequenced at a depth of 30X to broadly cover the whole genome, we have conservatively defined a variation as having a variant allele fraction (VAF) greater than 5% to enhance the robustness of our study. This threshold is higher than the 2%, or even 1.5%, typically referenced in previous study.^4^, ^7^, ^11^ These measures were taken to ensure that our identification of *DNMT3A*‐CHIP is both reliable and significant within the context of our research parameters. Furthermore, the clinical finding was verified in a mouse model in which inhibition of DNMT3A by RG108 increased infarct volume via proinflammatory pathways.

DNA methylation plays an important role in the ischemic injury of the brain and the regulation of inflammation.^36^ The level of total DNA methylation increases after ischemic stroke in mice, while the protein level of DNMTs is upregulated, mainly in DNMT3A, rather than DNMT1 or DNMT3b.^12^, ^17^ Genetic deletion of DNMT1 or treatment with the DNA methyltransferase inhibitor 5‐aza‐2′‐deoxycytidine intracerebroventricularly reduced the damage to ischemic tissue and infarct volume.^16^, ^37^ In contrast to the permanent and systemic changes introduced by knockout models, RG108 offers a temporal and potentially tissue‐specific approach that better mimics DNMT3A dysfunction. As *DNMT3A*‐CHIP is a pre‐existing condition in stroke patients, we administered the inhibitor once daily for 3 days prior to surgery and again at reperfusion onset to better simulate this clinical context. We found that DNMT3A inhibition by RG108 in a tMCAO stroke mouse model increased infarct volume and exacerbated neurobehavioral function, which is inconsistent with previous reports that inhibition of DNMTs function with antagonists or siRNA exerts neuroprotective effects.^13^, ^14^, ^15^, ^16^, ^17^, ^18^, ^19^ RG108 does not enter the brain at sufficient concentrations due to the blood‐brain barrier.^38^, ^39^ In previous studies, DNMTs inhibitors or siRNAs were incubated with primary cultured neurons or injected into the mouse brain by intracerebral stereotaxic injection, focusing on their protective effect in neurons.^13^, ^14^, ^15^, ^16^, ^17^, ^18^, ^19^ However, we selected different DNMTs inhibitors and injection methods, which focused on inflammation and yielded inconsistent results. Our results focused on how DNMT3A dysfunction affects the inflammatory immune cells after AIS and demonstrated that DNMT3A dysfunction causes neurological functional disability in AIS through the mechanism of neutrophil proliferation and infiltration. In a previous study that focused on the relationship between type 1 diabetes and stroke severity, Kalani et al.^12^ concluded that persistent systemic hyperglycemia exacerbates the inflammatory response after ischemic stroke and reduces the levels of DNMT1, DNMT3A, and global 5‐mC in the brain, leading to larger infarct size, more severe edema, and cell death.^12^ This is similar to our results to a certain extent, indicating a possible association between DNMT3A functional deficits via inflammation and poor functional outcomes after stroke. It reveals that DNMT3A inhibition can offer central neuroprotection, while peripheral DNMT3A inhibition increases inflammation and stroke severity. Abovementioned evidence underscores the complex roles of DNMT3A in epigenetic regulation and its systemic effects.

*DNMT3A* disruption in hematopoietic cells promotes cardiac dysfunction and macrophage accumulation in the myocardium, suggesting that hematopoietic *DNMT3A* deficiency leads to heart failure by upregulating specific inflammatory cytokines (such as IL‐6, CCL5, and CXCL1).^40^ In patients with severe degenerative aortic valve stenosis or chronic post‐ischemic heart failure, single‐cell RNA sequencing of peripheral blood monocytes showed that *DNMT3A*‐driven CHIP increased the expression of proinflammatory cytokines, cellular receptor CD163, and the NLRP3 inflammasome complex, although there was no significant difference in the plasma levels of IL‐6 and hsCRP between *DNMT3A*‐driven CHIP carriers and non‐CHIP carriers.^41^ It appears that the presence of *DNMT3A*‐driven CHIP causes an excessive inflammatory response in patients with cardiovascular disease.

Notably, we found that when DNMT3A function was inhibited, RNA‐seq results showed that most genes in the ischemic penumbra of stroke were upregulated, which is consistent with previous studies.^42^ The CXC family genes were significantly upregulated in the present study. Meanwhile, the upregulated DEGs were mainly enriched in proinflammatory biological processes, especially in the pathways of neutrophil migration and chemotaxis.^43^, ^44^, ^45^ However, previous studies using peripheral blood mononuclear cells have shown that aberrant DNA methylation could cause macrophage/monocyte dysfunction in atherosclerotic cardiovascular disease.^11^, ^42^, ^46^, ^47^, ^48^, ^49^ This discrepancy was probably due to the relatively small number of neutrophils during peripheral blood mononuclear cell preparation.^50^

Similarly, neutrophils are a key component of myeloid hematopoiesis in the immune inflammatory response after stroke.^51^ Neutrophils are recruited to the ischemic site within 24 h after stroke onset and reach a peak at 1−3 days, which worsens functional outcome.^52^, ^53^ Our findings showed that inhibition of DNMT3A function increased peripheral blood neutrophils and caused brain neutrophil infiltration after ischemic stroke, which is consistent with the RNA sequencing results. Previous studies have demonstrated that neutrophils are recruited to the infarct region, which can lead to the destruction of the blood‐brain barrier and impairment of vascular reconstruction and remodeling.^54^ Several studies investigating neutrophils as potential therapeutic targets have consistently demonstrated a reduction in neurological deficits and infarct volume. These interventions include depletion of polymorphonuclear neutrophils (PMNs) through administration of anti‐Ly6G antibodie^55^; inhibition of PMN brain entry via colchicine^56^ or CXCR2 or very late antigen‐4 blockade; and blocking of neutrophil adhesion to endothelial cells (intracellular adhesion molecule‐1 or selectins, etc.).^57^ Therefore, we illustrated that dysfunction of *DNMT3A* leads to the increase and infiltration of peripheral and central neutrophils and the excessive activation of proinflammatory processes, which in turn leads to a worse functional prognosis after stroke. It is necessary to explore targeted therapies for inflammatory pathways in the future.

One limitation of this study is that we mimicked the *DNMT3A* mutation in a mouse model by administering a DNMT3A inhibitor, which might not fully reflect the effects of the *DNMT3A* mutation. This approach has two known limitations: First, a small amount of RG108 may cross the blood–brain barrier (BBB) and exert a minor effect on neuronal DNMT3a; second, besides inhibiting DNMT3a, RG108 could also potentially affect other members of the DNMT family, such as DNMT1, which was not evaluated in this study. However, our study strongly suggests that DNMT3A dysfunction is associated with the risk of neurological functional disability by increasing neutrophil infiltration and the activation of proinflammatory factors. Second, it is regrettable that we did not observe neutrophil alterations in the laboratory indices of the clinical cohort. Given the complexity of clinical samples, it is necessary to design more rigorous cohorts in the future for further investigation. Third, our study focused on only one time point. Future studies may need to include more time points to clarify the overall effect of mutations on ischemic stroke.

In conclusion, our study provides evidence that DNMT3A dysfunction is associated with neurological functional disability in ischemic stroke patients and mice. The potential mechanism is an increase in neutrophil proliferation and infiltration into the ischemic brain region, resulting in proinflammatory activation and more damage to the brain tissue.

## 4 METHODS AND MATERIALS

### 4.1 Study population

Patients enrolled in this study were selected from the Third China National Stroke Registry (CNSR‐III),^20^ a nationwide prospective registry for patients presenting to hospitals with acute ischemic cerebrovascular events, including AIS and TIA. Patients from 201 hospitals across China were recruited for this study from August 2015 to March 2018. The diagnosis of AIS was based on the WHO criteria and confirmed by MRI or computed tomography of the brain. A total of 8524 AIS patients without a history of malignancy and recurrent stroke at 3 months were included in this study. More details on the patient enrollment process are shown in Figure S1. Signed informed consent was obtained from all the patients before they were enrolled in the study. The study protocol was approved by the ethics committee of Beijing Tiantan Hospital. Patients at 3‐month follow‐up visits were interviewed face‐to‐face by trained research coordinators. The mRS was used to evaluate the functional dependence of each patient (range 0 [no symptoms] to 6 [death]).^58^ The primary outcome in the current study was neurological functional disability, defined as an mRS score of 3−6.

### 4.2 Whole‐genome sequencing and somatic mutation detection

Samples preservation and WGS analysis protocols were published as previous.^21^ Total DNA was isolated from peripheral WBCs using a magnetic blood genomic DNA kit (DP329; TIANGEN Biotech Co Ltd). WGS was performed using the BGISEQ‐500 platform (BGI Genomics) with an average depth greater than 30× for each subject.^21^ GATK MuTect2 software (https://software.broadinstitute.org/gatk) was used to detect the putative somatic variants of *DNMT3A*. All variants with a VAF of at least 0.05 (5%) were considered. Variants that had been previously reported in the literature and/or the Catalog of Somatic Mutations in Cancer (http://cancer.sanger.ac.uk/cancergenome/projects/cosmic/) were used in the subsequent analysis.

### 4.3 Animals

Experiments were performed on C57Bl/6J male mice (age 8−10 weeks, weight 25 g). Animals were fed on a normal chow diet, had ad libitum access to food and water, and maintained at 24°C under a 12 h light/dark cycle.

Apart from G*Power, no other statistical methods were used to predetermine the sample size.^59^, ^60^ The number of experimental mice for each group is specified in the corresponding figure captions. The mice were randomized into each group and the investigators were blinded to allocation during experiments and outcome assessment.

### 4.4 Drug administration

RG108 (N‐phthalyl‐L‐tryptophan, DNA methylation inhibitor), 10 mg/kg (Selleck Chemicals; catalog number: S2821) or SGI‐1027, 10 mg/kg (Selleck Chemicals; catalog number: S7276) was dissolved separately in dimethyl sulfoxide and then diluted in normal saline. RG108/ SGI‐1027 and vehicle were injected via the tail vein once a day 3 days before surgery and once again at the onset of reperfusion, four times in total.

### 4.5 Cerebral ischemia model: transient middle cerebral artery occlusion

To induce ischemia/reperfusion brain injury, mice from both drug‐ and vehicle‐injected groups were subjected to tMCAO surgery, as previously described.^12^, ^61^ The tMCAO experiments were performed in a blinded manner. The detailed procedure of the tMCAO surgery can be found in the Supporting Information.

### 4.6 MR imaging of cerebral infarcts with mice

MRI scans were performed at the Beijing Neurosurgical Institute Imaging Facility using an echo planar capable, 7.0 T small animal MRI system (Bruker; BioSpec70/20USR). Scans were performed in anesthetized (2.5% ethobrom) mice 24 h poststroke, as previously described.^62^

### 4.7 Infarct analysis by TTC staining

At 24 h after cerebral ischemia, the mice were deeply anesthetized with isoflurane. The brains were rapidly removed and sliced into seven 1‐mm thick slices. The infarct volume was measured by TTC staining for approximately 30 min, as previously described.^12^ Using ImageJ software, we scanned and calculated the relative area of the corresponding region in each brain slice. The detailed calculating procedure of the infarct volume can be found in the Supporting Information.

### 4.8 Neurological function assessment

Mouse neurological function was assessed by experimenters without knowing any information about the mice. Briefly, we conducted a modified Garcia Test 18−24 h after tMCAO to assess neurological deficits, and five tests were performed, including body proprioception, vibrissae touch, limb symmetry, lateral turning, and forelimb walking. The scores on each test ranged from 0−3, as previously described.^24^, ^25^

### 4.9 Behavioral testing

All behavioral tests were carried out 24 h after tMCAO. The investigators were blinded to the allocation during the experiments and the outcome assessment.

Foot misplacement test: Mice were placed in a testing room for 60 min before testing. An automatic foot misplacement apparatus (Bioseb In Vivo Research Instruments; # BIO‐FMA) with a mouse corridor was used.

The open field test: Mice were placed in the open field from the same corner (50 cm × 50 cm × 40 cm, a total of 25 squares), after which they were adapted for 1 min. Their activities in the open field within 30 min were photographed and recorded.

The detailed procedure of the behavioral testing can be found in the Supplementary Information (SI).

### 4.10 RNA‐sequencing and data analysis

Mice were anesthetized 24 h after tMCAO and perfused with cooled phosphate‐buffered saline. The ischemic penumbra tissue was quickly dissected and separated on ice (the penumbra area was defined as adjacent to the infarct area, which was milky white after stripping the skull). Six mice were sampled (three in the vehicle group and three in the RG108 group). DEseq2^63^ was used for principal component analysis and identification of differentially expressed genes. Genes with an expression |log_2_fold change| > 1 and *q‐*value (adjusted *p*‐value) < 0.05 were defined as statistically significantly differentially expressed.

All heatmaps, volcanic maps, and violin diagrams were visualized using ggplot2 in R, version 4.1.0. The detailed procedure of the sample treatment and data analysis can be found in the Supporting Information.

### 4.11 ELISA assay

The levels of IL‐6 and 5‐mC in the peripheral blood were detected using IL‐6 (Abcam, ab222503) and 5‐mC (Epigentek; P1030) ELISA kits according to the manufacturer's instructions. Absorption at 450 nm was determined using a microplate reader (Tecan Trading AG; # SPARK). The concentration of IL‐6 and 5‐mC were determined according to the standard curve generated simultaneously.

### 4.12 Flow cytometry and hematological cell counts

Mouse peripheral blood and brain samples were collected and processed for single‐cell suspensions. Blood was drawn under deep anesthesia, and hematologic parameters were assessed using an automated cell counter machine (BC‐5000 Vet, Mindray Animal Medical). After blood collection, the brain was perfused and dissected, with the ischemic hemisphere treated with collagenase and digested at 37°C. The resulting mixture was centrifuged, and the pellet was resuspended and purified using Percoll gradient centrifugation to isolate viable cells. These were then resuspended in the FACS buffer for further analysis. A panel of fluorophore‐conjugated antibodies was used to label various immune cell types, including myeloid, microglia, neutrophil, and macrophage populations. The stained cells were analyzed using a Beckman flow cytometer (CytoFLEX S).

Flow cytometry data were subsequently analyzed using the Flow Jo software (BD, V10.8). Manual gating and subclustering of each sample was performed as indicated in the supplementary material (Figure S4), followed by unsupervised high‐dimensional subclustering analysis. The cell counts and the activated percentage of the indicated myeloid cell subgroups were calculated. The antibodies and reagents used in this study are listed in Table S2. The detailed procedure and additional data can be found in the Supporting Information.

### 4.13 Data analysis and statistics

#### 4.13.1 Clinical research

Categorical variables are presented as numbers (percentages). Continuous variables are presented as means with standard deviation or medians (IQR). Student's *t*‐test was used for continuous variables, and the chi‐square test was used for the comparison of response rate differences. The association of *DNMT3A* with a neurological functional disability was tested using logistic regression adjusted with an inverse probability of treatment weighting (IPTW) model. The IPTW model assigns weights to individuals based on their treatment probabilities, allowing for the estimation of causal effects in observational studies by addressing confounding. It creates a pseudopopulation with balanced treatment groups and provides an adjusted estimate of the treatment effect. Statistical significance was defined as a two‐sided *p*‐value < 0.05. Statistical analyses were conducted using SAS version 9.4 (SAS Institute Inc.).

#### 4.13.2 Basic scientific research

The number (*n*) of biological replicates or mice is indicated in the individual figure legends. The experimental variations in each graph are represented as the mean ± SEM. All the measurements were performed using independent samples. Independent Student's *t*‐test, one‐way analysis of variance (ANOVA), and repeated‐measurement two‐way ANOVA were employed for statistical analysis, as indicated in the individual figure legends. For multiple comparisons, ANOVA, post hoc Tukey, or Holm‐Sidak's tests were applied as indicated. Assumptions of normal data distribution and homoscedasticity were adopted in the *t*‐test and one‐way ANOVA. All statistical tests were two‐sided. Statistical significance was set at *p* < 0.05. The stack chart of mRS and all histogram charts were generated using GraphPad Prism (version 9.0.0).

## AUTHOR CONTRIBUTIONS

Zixiao Li and Yongjun Wang conceptualized and designed the study. Tian‐Jie Lyua, Xin Qiua, Yubo Wang, Ling Zhang, Yalun Dai, Xuechun Wang, Shunying Zhao, Meilin Xiang, Lu Cui, Si Cheng, Yang Liu, Hongqiu Gu, Yong Jiang, Yingyu Jiang, and Zhe Xu acquired and analyzed the data. Tian‐Jie Lyua, Xin Qiua, and Yubo Wang drafted the manuscript and the figures. Tian‐Jie Lyua, Xin Qiua, Yubo Wang, Yong Jiang, Xia Meng, Yilong Wang, Xingquan Zhao, Xianwei Wang, Qian Li, Meng Wang, Xinying Huang, Hao Li, Yongjun Wang, and Zixiao Li revised and approved the manuscript. All authors read and approved the final manuscript.

## CONFLICT OF INTEREST STATEMENT

The authors declare no conflict of interest.

## ETHICS APPROVAL

Patients were selected from the CNSR‐III study, which was approved by the ethics committee at Beijing Tiantan Hospital (IRB approval number: KY2015‐001‐01). Written informed consent was obtained from all participants. The Animal Welfare Ethics Committee of the Beijing Neurosurgical Institute approved the animal procedures (approval no. SYXK(Jing)2019‐0007;2020.12.1‐2023.12.1), which were conducted following the ethical principles outlined in the NIH Guide for the Care and Use of Laboratory Animals.

## Supporting information

Supporting Information

Supporting Information

## Data Availability

The raw RNA‐seq data of this study are available in the Genome Sequence Archive (GSA) repository (CRA009071). All data generated or analyzed during this study are included in this manuscript and its supplementary information files. Other data that support the findings of this study are available on reasonable request from the corresponding author.
