# Supplementary material for: DNMT3A dysfunction promotes neuroinflammation and exacerbates acute ischemic stroke
Source: MedComm (2020). 2024 Jul 14;5(7):e652. doi: 10.1002/mco2.652 (PMC11246610; doi:10.1002/mco2.652)
Supplement: Supplementary file 1 — Supporting Information [file MCO2-5-e652-s001.pdf]

Title Page

A short informative title

DNMT3A dysfunction promotes neuroinflammation and exacerbates acute ischemic stroke

A short running title

DNMT3A dysfunction and ischemic stroke

Authors

Tian-Jie Lyu[a,b,1], Xin Qiu[a,b,1], Yubo Wang[a,b,1], Ling Zhang[a], Yalun Dai[a,b], Xuechun Wang[a,b], Shunying Zhao[b], Meilin Xiang[b], Lu Cui[b], Si Cheng[a,b], Yang Liu[b], Hongqiu Gu[b], Yong Jiang[b], Xia Meng[a,b], Yilong Wang[a], Xingquan Zhao[a], Xianwei Wang[d], Qian Li[e], Meng Wang[b], Yingyu Jiang[b], Zhe Xu[b], Xinying Huang[b], Hao Li[b], Yongjun Wang[a,b,c,f,g,h,j,*], Zixiao Li[a,b,c,f,i,j,*]

a Department of Neurology, Beijing Tiantan Hospital, Capital Medical University, Beijing, China.
b China National Clinical Research Center for Neurological Diseases, Beijing, China.
c Clinical Center for Precision Medicine in Stroke, Capital Medical University, Beijing, China.
d Center for Metabolic Disease Research, Lewis Katz School of Medicine, Temple University, Philadelphia, PA, USA.
e Department of Biochemistry and Molecular Biology, School of Basic Medical Sciences, Capital Medical University, Beijing, China.
f Advanced Innovation Center for Human Brain Protection, Capital Medical University, Beijing, China.
g Research Unit of Artificial Intelligence in Cerebrovascular Disease, Chinese Academy of Medical Sciences, Beijing, China.
h Center for Excellence in Brain Science and Intelligence Technology Shanghai, Chinese Academy of Sciences, Shanghai, China.
i Chinese Institute for Brain Research, Beijing, China.
j Beijing Engineering Research Center of Digital Healthcare for Neurological Diseases, China.
[1] Tian-Jie Lyu, Xin Qiu, and Yubo Wang contributed equally to this work.
[*]Yongjun Wang and Zixiao Li jointly supervised this work.

†Correspondence

Yongjun Wang, PhD, MD (yongjunwang@ncrcnd.org.cn) and Zixiao Li, PhD, MD (lizixiao2008@hotmail.com). Department of Neurology, Beijing Tiantan Hospital, Capital Medical University. No. 119 South 4th Ring West Rd, Fengtai District. Chinese Institute for Brain Research, Beijing, China, 100070.

Funding information

National Natural Science Foundation of China, Grant/Award Number: 82171270, 82111530203 and U20A20358); Beijing Natural Science Foundation, Grant/Award Number: Z200016; Ministry of Science and Technology of the People's Republic of China, Grant/Award Number: 2017YFC1310901 and 2016YFC0901002; CAMS Innovation Fund for Medical Sciences, Grant/Award Number: 2019-I2M-5-029.

1. Supplementary Methods

1.1 Patient clinical information and imaging data

The clinical data of all patients were prospectively collected. The measurement of laboratory biomarkers has been previously described[20]. Blood samples were collected using EDTA anticoagulation tubes. Hs-CRP, hemoglobin (HGB), WBC, neutrophil (NEUT), triglyceride (TG), low-density lipoprotein (LDL), high-density lipoprotein (HDL), and cholesterol (CHOL) levels were measured in plasma samples using standard hospital assays. IL-6 and interleukin-1 receptor antagonist (IL-1RA) levels were measured centrally in Beijing Tiantan Hospital.

All patients recruited in the CNSR-III were recommended to undergo brain MRI (including T1-/T2-weighted, diffusion-weighted imaging [DWI] with apparent diffusion coefficient, fluid-attenuated inversion recovery [FLAIR], and magnetic resonance angiography [MRA]) and at least one assessment of intracranial arteries (including MRA, computed tomography angiography [CTA], or digital subtraction angiography [DSA]). The degree of intracranial artery stenosis (ICAS) was assessed by trained experts according to the Warfarin- Aspirin Symptomatic Intracranial Disease Study criteria[59].

1.2 Cerebral ischemia model: transient middle cerebral artery occlusion (tMCAO)

To induce ischemia/reperfusion brain injury, mice from both drug- and vehicle-injected groups were subjected to tMCAO surgery, as previously described[12,62]. First, mice were anesthetized with 4% isoflurane and maintained on 1.5% isoflurane. Under an operating microscope, the right common carotid artery (CCA), external carotid artery (ECA), and

internal carotid artery (ICA) were carefully exposed after a midline cervical incision. Subsequently, a silicone-coated monofilament (Doccol Corporation, Sharon, MA, USA; 602056PK10) with a diameter of 0.20 mm was inserted into the ICA via a slit on the ECA and advanced 9–12 mm from the common carotid bifurcation. The inserted embolus was temporarily immobilized and ischemia was maintained for 60 min. The monofilament was then removed and wound care was administered. Animals were carefully monitored and cared for 24 h. The same procedure was performed for sham-operated animals. However, the sham group only separated the ICA, ECA, and CCA without interruption of cerebral blood flow in the middle cerebral artery. The tMCAO experiments were performed in a blinded manner.

1.3 Infarct analysis by TTC staining

At 24 hours after cerebral ischemia, the mice were deeply anesthetized with isoflurane. The brains were rapidly removed and sliced into seven 1-mm thick slices. The infarct volume was measured by TTC staining for approximately 30 min, as previously described[12]. Using ImageJ software, we scanned and calculated the relative area of the corresponding region in each brain slice. We employed an automated system for selecting the region of interest (ROI) and calculating the relative infarct area in brain slices as previously reported by Chan *et al.* (2020)[64]. This system first segments the mouse brain slice from the image, draws the cerebral central line to divide it into contralateral and ipsilateral hemispheres, then segments the infarct from the ipsilateral hemisphere using gamma correction and thresholding, and finally extracts the corpus callosum from the contralateral hemisphere. The infarct volume (S1) is computed based

on the segmented areas, providing an automated and reliable method for our analysis. The infarction rate for each mouse was calculated as:

Infarct volume (%) = [Σ(S1-S2)]/Σ2S1, where S1 represents the half-brain area of the healthy side, S2 represents the normal part of the affected side, and '2S1' represents the area of the intact corresponding brain slice before injury.

1.4 Behavioral testing

All behavioral tests were carried out 24 h after tMCAO. The investigators were blinded to the allocation during the experiments and the outcome assessment.

Foot misplacement test: Mice were placed in a testing room for 60 min before testing. An automatic foot misplacement apparatus (Bioseb In Vivo Research Instruments, Pinellas Park, FL, USA; # BIO-FMA) with a mouse corridor was used. The corridor floor had ladder rungs, and the mice walked on the ladder rungs. Sensors identify the number of times the paws of mice slip through the ladder rungs. Locotronic software (Bioseb In Vivo Research Instruments) was used to record the number of missed steps. Mice were placed on a track composed of discontinuous sticks. At the beginning of the experiment, the mice were stimulated by the entrance to the exit. The device automatically recorded the number of limb treads during the movement of the mice until the experiment was terminated automatically after moving 50 cm. If a mouse changed direction midway through the recording, the walk was repeated until the mouse walked the full length of the corridor without changing direction. The mice were allowed to walk back through the corridor to the original starting point. Missed steps on the return walk were not recorded. This back-and-forth walk was repeated three

times, for a total of four back-and-forth walks. The first walk served as “practice” and was excluded from the analysis. Three recordings per mouse were averaged to obtain the number of missed steps per mouse[26,27,65].

The open field test: Mice were placed in the open field from the same corner (50 cm × 50 cm × 40 cm, a total of 25 squares), after which they were adapted for 1 min. Their activities in the open field within 30 min were photographed and recorded, including the square number of horizontal movements (four paws into the grid was considered as one time) and the vertical standing condition (two paws in the air and then putting them down was considered as one time). Autonomous activity frequency was the sum of all conditions.

1.5 RNA-sequencing and data analysis

Mice were anesthetized 24 h after tMCAO and perfused with cooled phosphate-buffered saline (PBS). The ischemic penumbra tissue was quickly dissected and separated on ice (the penumbra area was defined as adjacent to the infarct area, which was milky white after stripping the skull). Six mice were sampled (three in the vehicle group and three in the RG108 group). Tissues were immediately frozen in liquid nitrogen. The samples were then submitted to the cooperative company Berry Genomics for subsequent RNA extraction, cDNA library preparation, and RNA sequencing. Total RNA was quantified as follows: (1) RNA purity and concentration were examined using a NanoDrop 2000, and (2) RNA integrity and quantity were measured using the Agilent 2100/4200 system. The RNA library for RNA-seq was prepared as follows: mRNA was purified from total RNA using poly-T and then

fragmented into 300–350 bp fragments, and the first strand cDNA was reverse-transcribed using fragmented RNA and dNTPs (dATP, dTTP, dCTP, and dGTP), and second strand cDNA synthesis was subsequently performed. The remaining overhangs of double-stranded cDNA were converted into blunt ends via exonuclease/polymerase activity. After adenylation of the 3' ends of the DNA fragments, sequencing adaptors were ligated to the cDNA, and the library fragments were purified. The template was enriched by PCR and the PCR product was purified to obtain the final library. After library construction, library concentration was measured using a Qubit® fluorometer. The accurate concentration of the cDNA library was determined using qPCR. The size distribution of the library was determined using agarose gel electrophoresis. After library preparation and pooling of the different samples, the samples were subjected to Illumina sequencing. Commonly, lncRNA-seq uses PE150 (paired-end 150nt) sequencing. Raw data (raw reads) in the FASTQ format were first processed using in-house Perl scripts. In this step, clean data (clean reads) were obtained by removing the following reads: (1) reads with adapter, (2) reads with more than 3 N, and (3) reads with more than 20% nucleotides with Qphred<=5; At the same time, Q20, Q30, and GC content of the clean data were calculated. The clean reads were then mapped to the Silva database to remove rRNA. All downstream analyses were based on clean data without rRNA. Paired-end clean reads were aligned to the reference genome using Hisat2[66]. Feature count[67] was used to count the read numbers mapped to each gene.

DEseq2[68] was used for principal component analysis (PCA) and identification of differentially expressed genes (DEGs). Genes with an expression $|\log_2 \text{fold change}|>1$

and q-value (adjusted P-value) <0.05 were defined as statistically significantly differentially expressed. We sorted all upregulated DEGs by q-value and collected ten significantly expressed genes to construct the gene-GO network. ClusterProfler[69] is an R package used for enrichment analysis. Using clusterprofer, we used biological processes in the GO[70] and KEGG[71] to analyze the upregulated DEGs. GSEA[28] was used for the enriched pathway analysis from the GO and KEGG analyses with all upregulated and downregulated DEGs.

We calculated the fragments per kilobase of gene model per million mapped reads (FPKM) values using the TPMCalculator[72]. All heatmaps, volcanic maps, and violin diagrams were visualized using ggplot2 in R, version 4.1.0.

1.6 Western blot

For western blotting, brain tissues were sampled 24 hours after tMCAO. Briefly, tissue was lysed (RIPA: PMSF=100:1) and centrifuged at 12,000 rpm and 4℃ for 20 min to collect the supernatant. Protein concentration was measured using a bicinchoninic acid (BCA) protein assay kit (Thermo Fisher Scientific, 23225). The protein supernatant was added to 1x SDS protein loading buffer with bromophenol blue (APPLYGEN, B1012-5), heated at 95–100℃ for 10 min, and centrifuged at 12,000 rpm for 2 min. The supernatant was collected for loading. Protein samples (25–30 μg total protein) were resolved with precast SDS-PAGE gel (4-20% Bis-Tris MOPS 15-Well; APPLYGEN, Beijing, China; T202110) and probed with the primary antibody DNMT3a (Abcam, Cambridge, UK; ab2850, 1:1,000) and GAPDH (ZSGB-BIO, Beijing, China; TA-08, 1:2,000).

1.7 Quantitative Reverse Transcription PCR (RT–qPCR)

For RT-qPCR, total RNA was extracted from the brain tissues using the EASYspin tissue/cell total RNA extraction kit (Aidlab Biotechnologies, Ltd., Beijing, China; RN2802). Total RNA was reverse transcription with 5X All-In-One RT MasterMix (Applied Biological Materials Inc., Richmond, BC, Canada; G490). RT–qPCR was performed using a miScript SYBR Green PCR Kit (Thermo Fisher Scientific, Waltham, MA, USA; A25741) on a Step-One Plus PCR instrument (Applied Biosystems, Waltham, MA, USA). Primers used in this study are listed in **Supplementary Table S1.**

1.8 Flow cytometry and Hematological cell counts

Mouse peripheral blood and brain single-cell suspensions were prepared as described previously[73,74]. Briefly, mouse peripheral blood was harvested through the angular vein using an anticoagulant tube after deep anesthesia. Peripheral blood samples were analyzed for hematologic parameters using an automated cell counter machine (BC-5000 Vet, Mindray Animal Medical, Mahwah, NJ, USA). After peripheral blood collection, transcranial perfusion was performed using precooled 0.9% NaCl. Brain tissue was dissected and placed in PBS on ice. The ischemic hemisphere (MCAO ipsilateral brain) was placed in 1 ml of collagenase I (1 mg/ml, C8140, Solarbio, Beijing, China) preheated at 37℃, immediately dissected, and minced into pieces. The samples were transferred to a low-speed rotating hybrid furnace and incubated at 37℃ for 30 min digestion. The digested mixture was transferred to a new 15 ml centrifuge tube, and 2 ml PBS was added to terminate the digestion. The mixture was blown 10–20

times until no large debris was visible. Then the mixture was centrifuged at 2,000 rpm 4℃ for 10 min, and the supernatant was discarded. The 30% Percoll was added to resuspend the pellets, followed by centrifugation at 700 g for 10 min at 4℃. The myelin debris fraction floated on the surface and was carefully poured out, then the pelleted cells were resuspended by 5 mL PBS and centrifuged at 350 g for 10 min at 4℃ to remove the residual Percoll. Then, 1 ml FACS buffer (1 x PBS with 1% BSA) was added to resuspend the pelleted cells, and 1.0 x 10^6 cells per tube were collected for subsequent use.

The Zombie UV™ Fixable Viability Kit (423108, BioLegend, San Diego, CA, USA) was used to discriminate between live and dead cells. The primary antibody of CXCR2 (bs-4836R, Bioss, Woburn, MA, USA), followed by a secondary antibody labeled with Alexa Fluor™ Plus 594 (A32754, Invitrogen, Waltham, MA, USA) was used to label the activated immune cells. A panel of fluorophore-conjugated antibodies containing CD45-APC/Cy7, F4/80-FITC, CD11b-PE/Cy7, and Ly6G-PE was used for myeloid/microglia /neutrophil/macrophage labeling. All the antibody incubations were as follows: cells were incubated with antibody in a 100 μl volume on ice for 30 min, then added 1 ml FACS buffer (1 x PBS with 1% BSA) was followed by centrifugation at 350 g for 10 min at 4℃. Finally, pre-stained cells were analyzed using a Beckman flow cytometer (Brea, CA, USA; CytoFLEX S).

Flow cytometry data were subsequently analyzed using the Flow Jo software (BD, V10.8). Manual gating and sub-clustering of each sample were performed as indicated in the supplementary material (**Supplementary Figure S4**), followed by unsupervised high-dimensional sub-clustering analysis. Myeloid cells ($CD45^{+}CD11b^{+}$) from all samples (5,500 cells per sample) were concatenated to perform sub-clustering with high-dimensional analysis. The opt-SNE[75] and FlowSom[76] unsupervised clustering algorithms were used for visualization and sub-clustering. FlowSom clusters were merged using the matched MFI values, and the sub-clusters were gated manually based on FlowSom clustering. The cell counts and the activated percentage of the indicated myeloid cell subgroups were calculated. The antibodies and reagents used in this study are listed in **Supplementary Table S2.**

2. Supplementary figures and legends

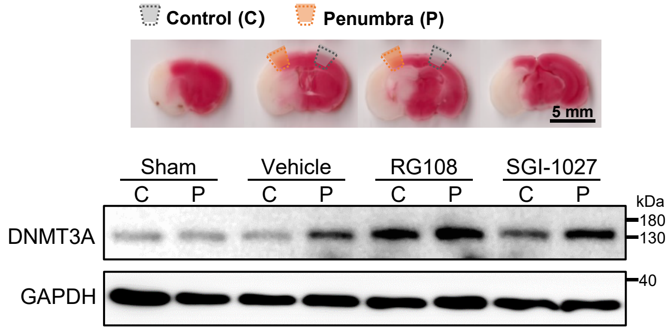

Figure S1. RG108 shows superiority over SGI-1027 in DNMT3A functional inhibition.

Brain section with penumbra (upper) and western blot for DNMT3A in the infarct penumbra and contralateral region (lower).

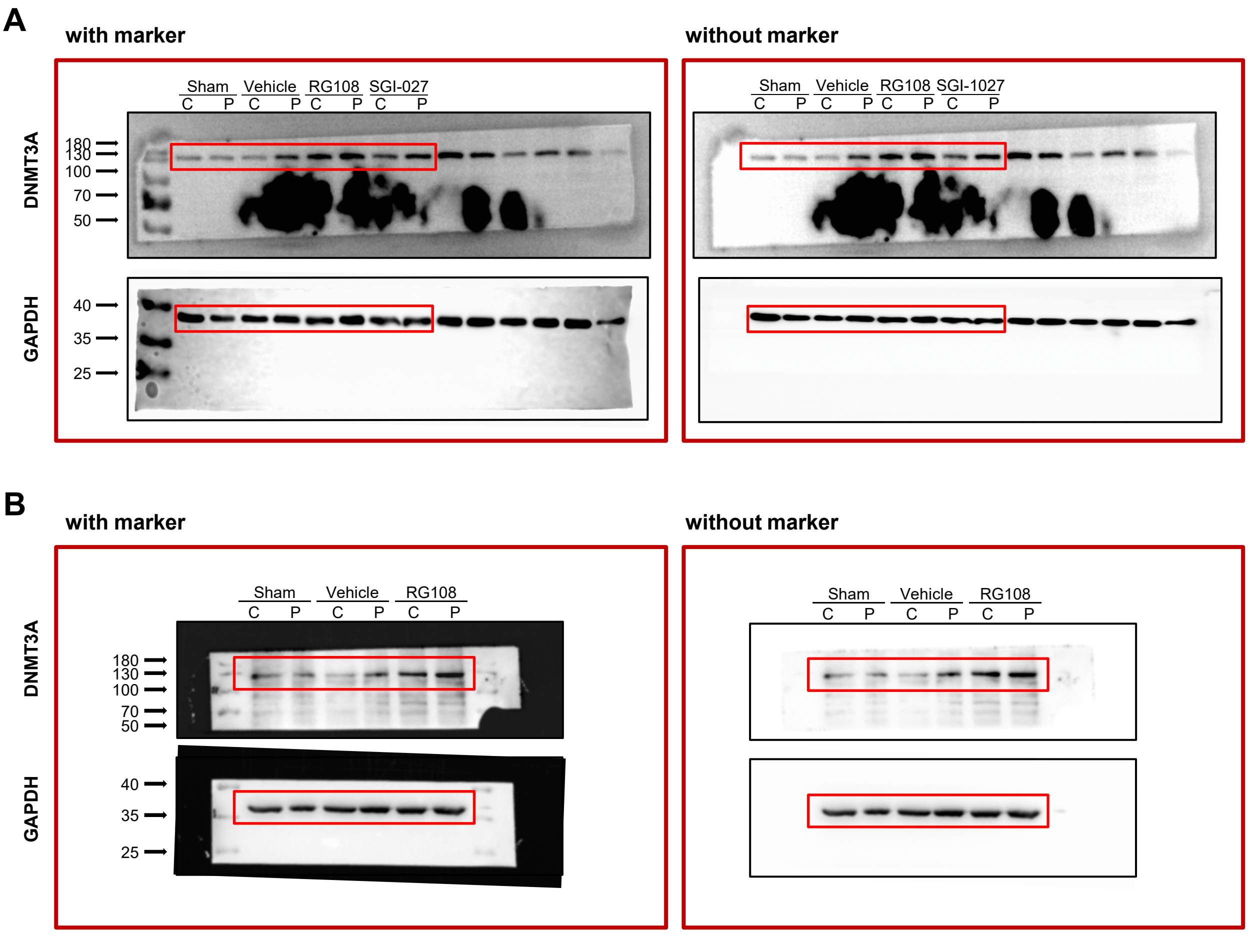

Figure S2. Raw images of the western blot in Figure S1 (upper, A) and Figure 5A (down, B).

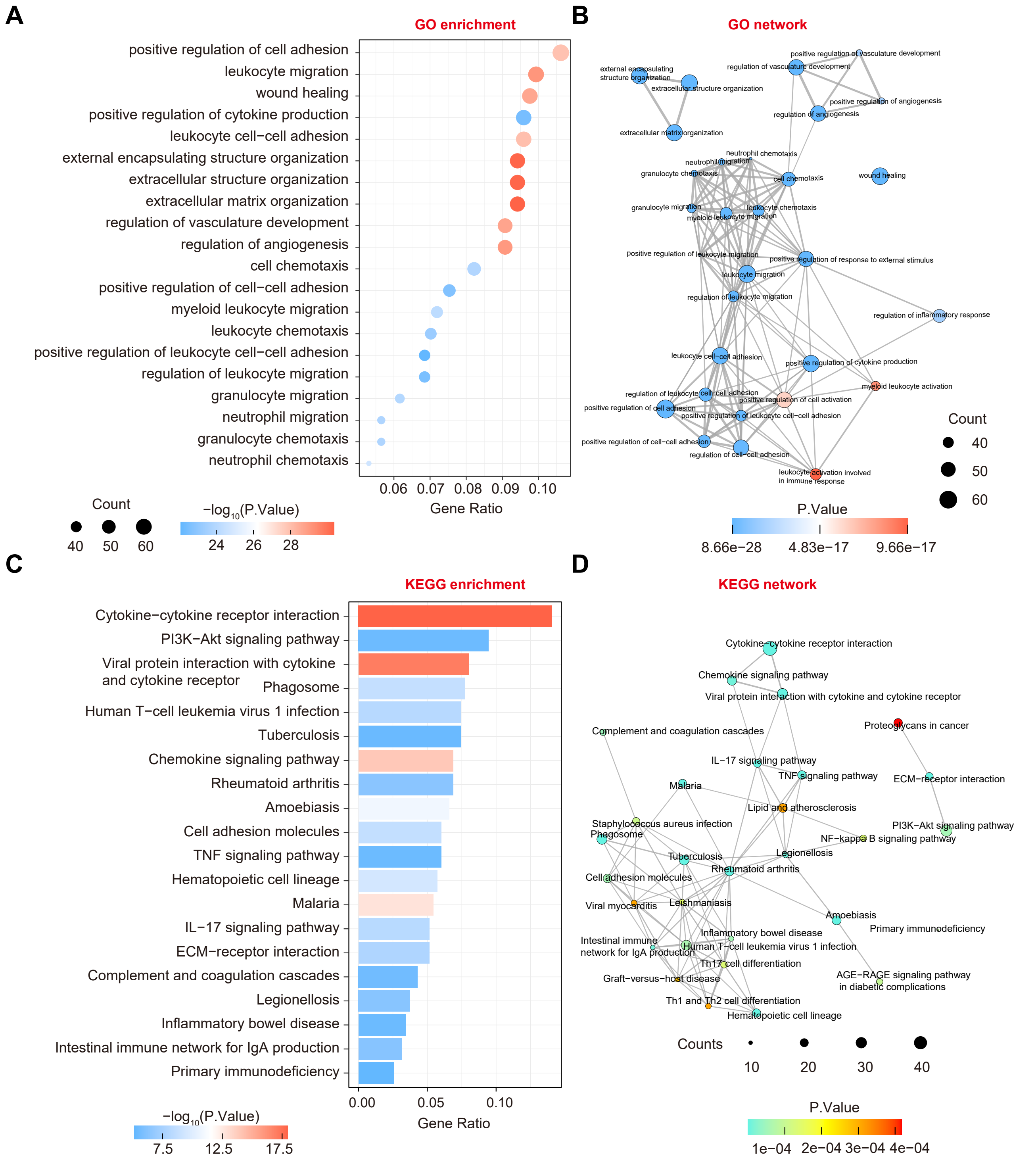

Figure S3. Enrichment analysis of all the 616 up-regulated DEGs.

A-D All 616 upregulated DEGs were used for enrichment analysis to identify the most significantly up-regulated molecular function and pathways, including biological process in the GO enrichment **(A),** GO network by enrichplot (R package) **(B)**, KEGG signal pathway **(C)** and KEGG network by clusterProfiler (R package) **(D)**; representative gene counts and *P*-values were shown as legends indicated.

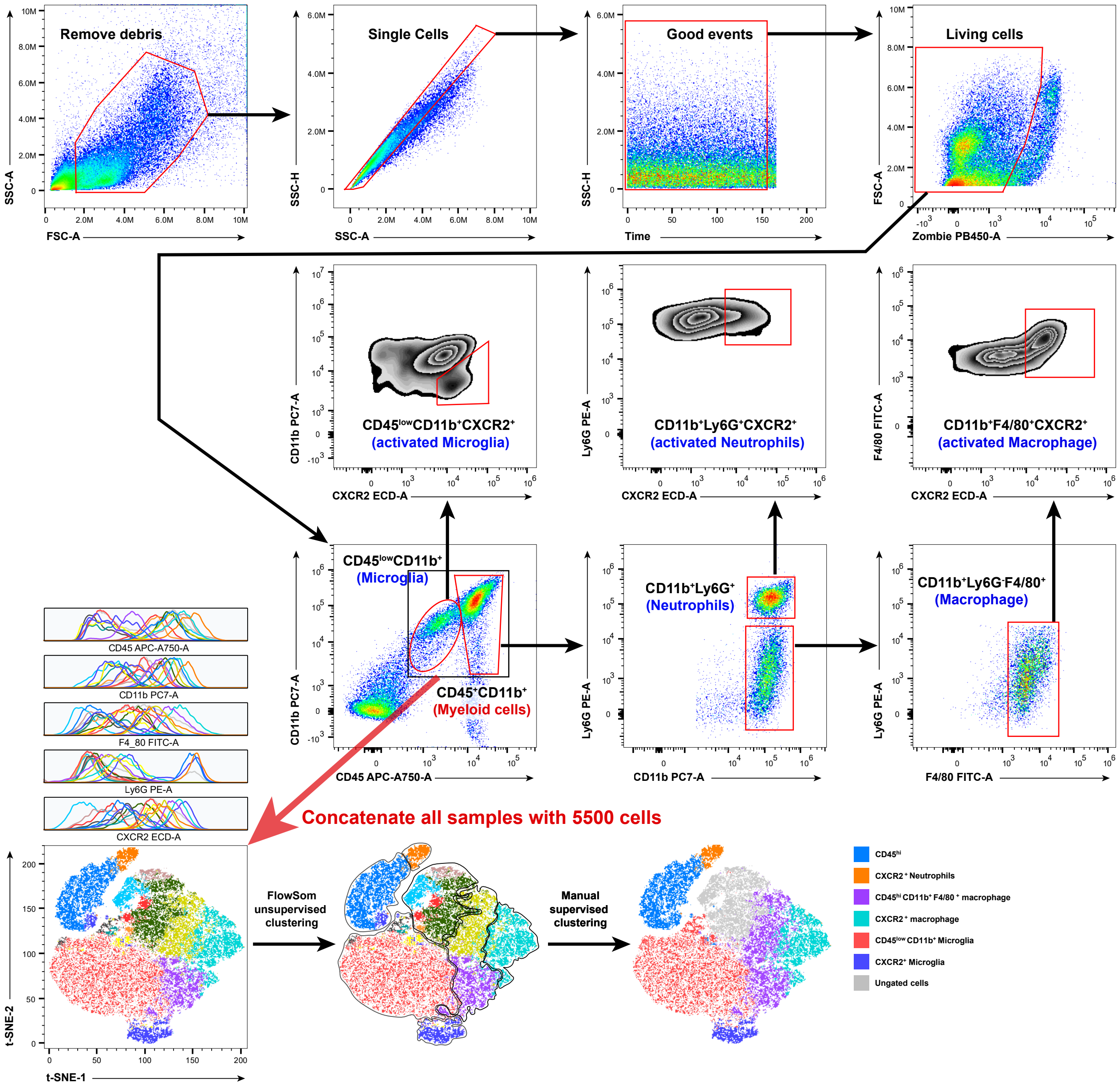

Figure S4. Gating and sub-clustering strategy of flow cytometry with high-dimensional analysis for poststroke mouse brain cells.

The ipsilateral brain hemisphere was collected from each mouse 24 hours after tMCAO. Single-cell suspensions were made for immunofluorescence staining of immune cell surface markers and flow cytometry was performed. Fluorescence minus one (FMO) control was used in all gates. Debris was excluded based on FSC-A/SSC-A scattering; Doublets were excluded to discriminate singlets based on SSC-H/SSC-A scattering; Bad events were excluded when fluctuations occurred during a period of collecting time; Dead cells were excluded with Zombie UV™ Fixable Viability Kit. The immune cells in the brain were gated out as follows: myeloid cells (CD45^{+}CD11b^{+}), microglia (CD45^{low}CD11b^{+}), neutrophils (CD45^{+}CD11b^{+}Ly6G^{+}), macrophage (CD45^{+}CD11b^{+}Ly6G^{-}F4/80^{+}). Besides, CXCR2 was used as a marker of activated myeloid cells. Myeloid cells (CD45^{+}CD11b^{+}) of all samples (5500 cells per sample) were concatenated to perform sub-clustering with high-dimensional analysis. Firstly, the opt-SNE algorithm was used for visualization of dimensionality reduction analysis and formed a t-SNE map; Secondly, hierarchical sub-clustering was performed with FlowSom (number of meta-clusters=16),

which was an unsupervised clustering algorithm, and projected onto the t-SNE map; Expression of each marker was measured as the corresponding histogram upper the t-SNE map; Thirdly, FlowSom clusters were merged by the matched MFI values and the subclusters were gated manually based on the FlowSom clustering.

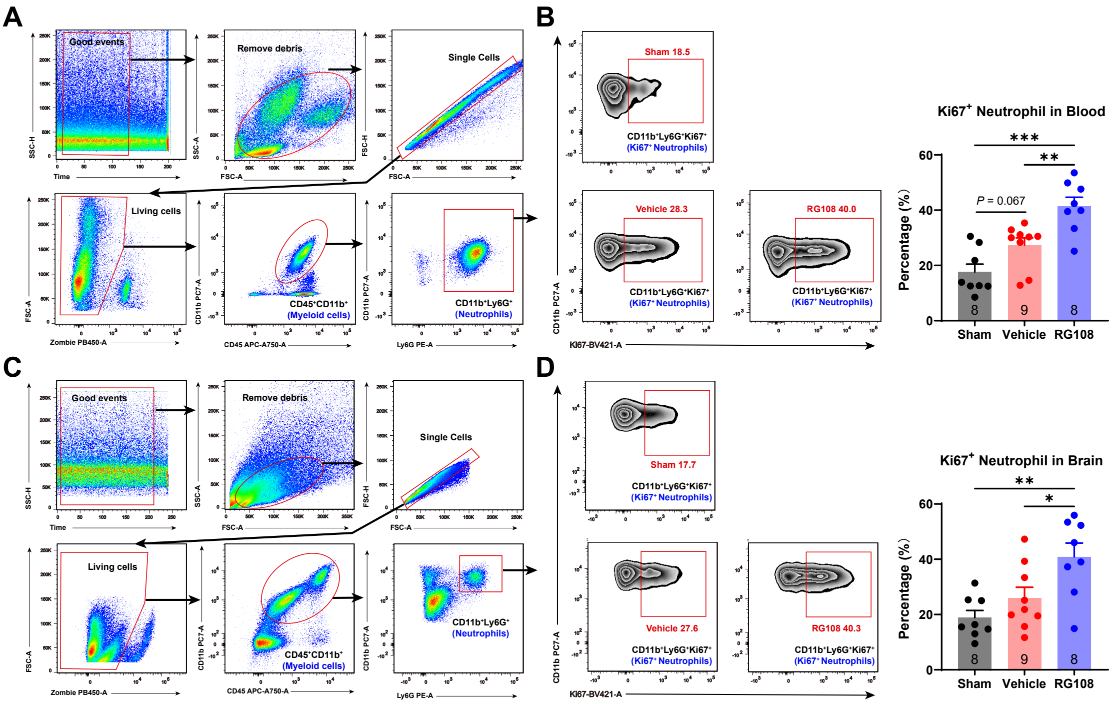

Figure S5. DNMT3A inhibition by RG108 leads to an increase in Ki67-positive neutrophils in peripheral blood and ischemic brain tissue.

A Gating strategy for the identification of neutrophils in peripheral blood samples using flow cytometry. **B** Proportion of Ki67+ neutrophils in peripheral blood. Representative flow cytometry plots are shown on the left, and quantitative analysis is presented on the right as statistical graphs. **C** Gating strategy for infiltrating neutrophils in the ischemic hemisphere using flow cytometry. **D** Proportion of Ki67+ infiltrating neutrophils in the ischemic hemisphere. Representative flow cytometry plots on the left with corresponding statistical analysis on the right. Sham, n=8; Vehicle, n=9; RG108, n=8. One-way ANOVA with Tukey's multiple comparisons post hoc test was used. *p < 0.05，**p < 0.01, ***p < 0.001, All data were expressed as mean±SEM with individual plots.
